# Analytical and clinical performance of in-house and commercial real-time PCR assays for diagnosing *L. infantum* visceral leishmaniasis: a study from a hub center in Northern Italy

**DOI:** 10.1128/jcm.01084-25

**Published:** 2026-02-13

**Authors:** Tommaso Gritti, Beatrice Mola, Lisa Argnani, Bianca Granozzi, Michele Bacchiega, Fraia Melchionda, Arianna Dondi, Giacomo Nigrisoli, Silvia Castaldini, Luca Prodi, Tiziana Lazzarotto, Matt Denwood, Stefania Varani, Margherita Ortalli

**Affiliations:** 1 Department of Medical and Surgical Sciences, University of Bologna 198207, Bologna, Italy; 2 Infectious Diseases Unit, IRCCS Azienda Ospedaliero-Universitaria di Bologna, Bologna, Italy; 3 Pediatric Hematology and Oncology, IRCCS Azienda Ospedaliero-Universitaria di Bologna, Bologna, Italy; 4 Pediatric Emergency Unit, IRCCS Azienda Ospedaliero-Universitaria di Bologna, Bologna, Italy; 5 Department of Chemistry ‘‘Giacomo Ciamician’, University of Bologna 9296, Bologna, Italy; 6 Preclinical and Traslational Research in Oncology-PRO, IRCCS Azienda Ospedaliero-Universitaria di Bologna, Bologna, Italy; 7 Unit of Clinical Microbiology, IRCCS Azienda Ospedaliero-Universitaria di Bologna, Bologna, Italy; 8 Department of Veterinary and Animal Sciences, Faculty of Health and Medical Sciences, University of Copenhagen 555025, Frederiksberg, Denmark; Mayo Clinic Minnesota, Rochester, Minnesota, USA

**Keywords:** visceral leishmaniasis, kinetoplast minicircle DNA, real-time PCR, *Leishmania infantum*, molecular diagnosis

## Abstract

Molecular methods are increasingly used to diagnose visceral leishmaniasis (VL), a life-threatening disease caused by *Leishmania infantum* in the Mediterranean basin. In this study, we compared the analytical and clinical performances of three real-time polymerase chain reaction (PCR) assays used for VL diagnosis at the University Hospital of Bologna, Northern Italy. The first test, which is commercially available, targets the small subunit of the 18S ribosomal RNA gene of *Leishmania* (rDNA). The second and third methods, developed in-house, target the rDNA and the kinetoplast minicircle conserved region (kDNA). The three PCR assays were performed on standard samples prepared using a *L. infantum* reference strain, as well as on 90 peripheral blood samples from patients with clinical suspicion of VL. Among these, 33 were confirmed as VL cases. The in-house kDNA PCR exhibited the highest observed analytical and clinical sensitivity. Conversely, kDNA PCR showed lower specificity (95%) on clinical samples when compared to rDNA amplification assays (100%). The three methods exhibited excellent concordance in VL identification (Cohen’s Kappa ≥ 0.9). The higher sensitivity of the kDNA target compared to the rDNA target was confirmed by analyzing the cycle threshold values obtained from parasitic DNA amplification using the three real-time PCR assays in VL-positive samples. In summary, all three molecular assays exhibited good analytical and clinical performance, with the kDNA-based PCR showing the highest sensitivity. These findings support kDNA as the most suitable target for detecting low levels of *Leishmania* DNA in peripheral blood samples from VL patients.

**IMPORTANCE:**

Visceral leishmaniasis is a life-threatening disease, rendering early and accurate diagnosis essential for patient survival. However, highly sensitive diagnostic tools are lacking. In this study, we compared the analytical and clinical performances of three real-time PCR assays routinely used for diagnosis of visceral leishmaniasis in a referral center in Northern Italy. All three assays exhibited robust diagnostic performance, with the PCR targeting the conserved region of the kinetoplast minicircle DNA exhibiting higher sensitivity than the assays targeting the small subunit of the 18S ribosomal RNA gene. This enhanced sensitivity is crucial for detecting visceral leishmaniasis in patients with low concentration of parasitic DNA in peripheral blood, as misdiagnosis in these patients can lead to severe consequences. Our findings highlight the need for the development of commercial, automated assays targeting the kinetoplast minicircle DNA to enhance the accuracy of the diagnosis of this potentially lethal disease.

## INTRODUCTION

Visceral leishmaniasis (VL) is a systemic infectious disease caused by hemoflagellate protozoa of the genus *Leishmania*, mostly by species within the *Leishmania donovani* complex, i.e., *Leishmania infantum* and *L. donovani* (1). The disease is transmitted by blood-feeding vectors of the subfamily *Phlebotominae* (order Diptera), known as sand flies.

VL is endemic in southern Europe, where it is caused by *L. infantum* (2). In this region, the disease is emerging or re-emerging in localized foci of infection (3). In recent years, Italy has reported an increase of VL cases in some specific areas; a multi-annual outbreak with fluctuating number of cases has been ongoing in the Metropolitan city of Bologna in Northern Italy from 2010 (4), while a more recent re-emergence of human cases was observed in Tuscany, Central Italy (5).

VL results from the systemic dissemination of the *Leishmania* parasites (1). Replication of amastigotes occurs within macrophages, which spread the infection through the bloodstream to the spleen, liver, and bone marrow, leading to spleen and liver enlargement as well as pancytopenia (6). Other common clinical features of VL include irregular and prolonged fever and weight loss. However, these signs are non-specific, and a definitive diagnosis requires confirmation through parasitological and/or serological tests (1, 2).

In particular, serological tests are usually the first-line tests (2), but they cannot distinguish between active, past, or asymptomatic infections, and their performance varies depending on the antigen used, the geographic region, and patient’s immunological status (2, 7, 8).

Currently, no universally accepted gold standard exists for the diagnosis of VL beyond the microscopic examination or isolation of parasites from bone marrow or splenic aspirates (2). These techniques require invasive procedures for sampling and exhibit sensitivities of >90% for spleen microscopy and 53%–86% for bone marrow microscopy, while parasite culture has a sensitivity of 60%–85% (1, 9). The suboptimal sensitivity of traditional techniques poses a significant challenge in VL diagnosis, as the disease is fatal if not promptly identified and treated.

Polymerase chain reaction (PCR)-based methods have become extensively used to diagnose VL, especially in high-income countries. Two systematic reviews have reported high pooled sensitivity (>85%) and specificity (>95%) for real-time PCR assay using peripheral blood (10, 11). In addition, quantitative PCR (qPCR) assays can evaluate the concentration of parasitic DNA in VL patients, thus allowing to monitor the treatment outcome (12, 13). These methods target different genomic segments of *Leishmania* spp., with a particular focus on multi-copy genes that are characteristic of this genus, thus enhancing sensitivity (12).

Among the targets used for molecular diagnosis, two are of particular interest: the gene coding for the small subunit of the 18S ribosomal RNA gene (rDNA) located on chromosome 27 and the kinetoplast minicircle DNA (kDNA). The rDNA is a multi-copy gene, present in approximately 20–40 copies per parasite, and it is highly conserved in the *Leishmania* genus (14). In addition, the kDNA is a mitochondrial structure found exclusively in protozoa of the order *Kinetoplastidia*, which includes the genus *Leishmania* (15). It consists of a dense network of interlocked circular DNA molecules in the kinetoplast, referred to as minicircles and present in thousands of copies per parasitic cell.

Although PCR is widely considered more sensitive than microscopy and culture and qPCR is a valuable tool for monitoring VL after treatment (2, 16), standardized protocols are lacking, and no consensus exists regarding the optimal genetic target for amplification. Furthermore, comparative studies evaluating the performance of in-house and commercial PCR assays for VL diagnosis remain limited (17). The aim of the present study was to evaluate the diagnostic performance of real-time PCR assays used for VL diagnosis at the Regional Reference Laboratory (RRL) for leishmaniasis within the Microbiology Unit of the University Hospital of Bologna, Northern Italy.

## MATERIALS AND METHODS

### Study design

This is a diagnostic accuracy study; we compared the performance of three real-time PCR assays used for VL diagnosis at the RRL, University Hospital of Bologna, Italy. The first method, which is commercially available, is distributed by Clonit Srl and carried out on the semi-automated extraction-amplification ELITe InGenius SP200 equipment. This kit targets the *Leishmania* rDNA sequence and is referred in this study as Clonit PCR. The second and third methods, developed in-house, target two distinct *Leishmania* DNA sequences: the rDNA and the kDNA region, respectively. The primers and probes for the in-house PCR assays were designed by Wortmann et al. (14) for the rDNA target and by Mary et al. for the kDNA target (18).

**Fig 1:**
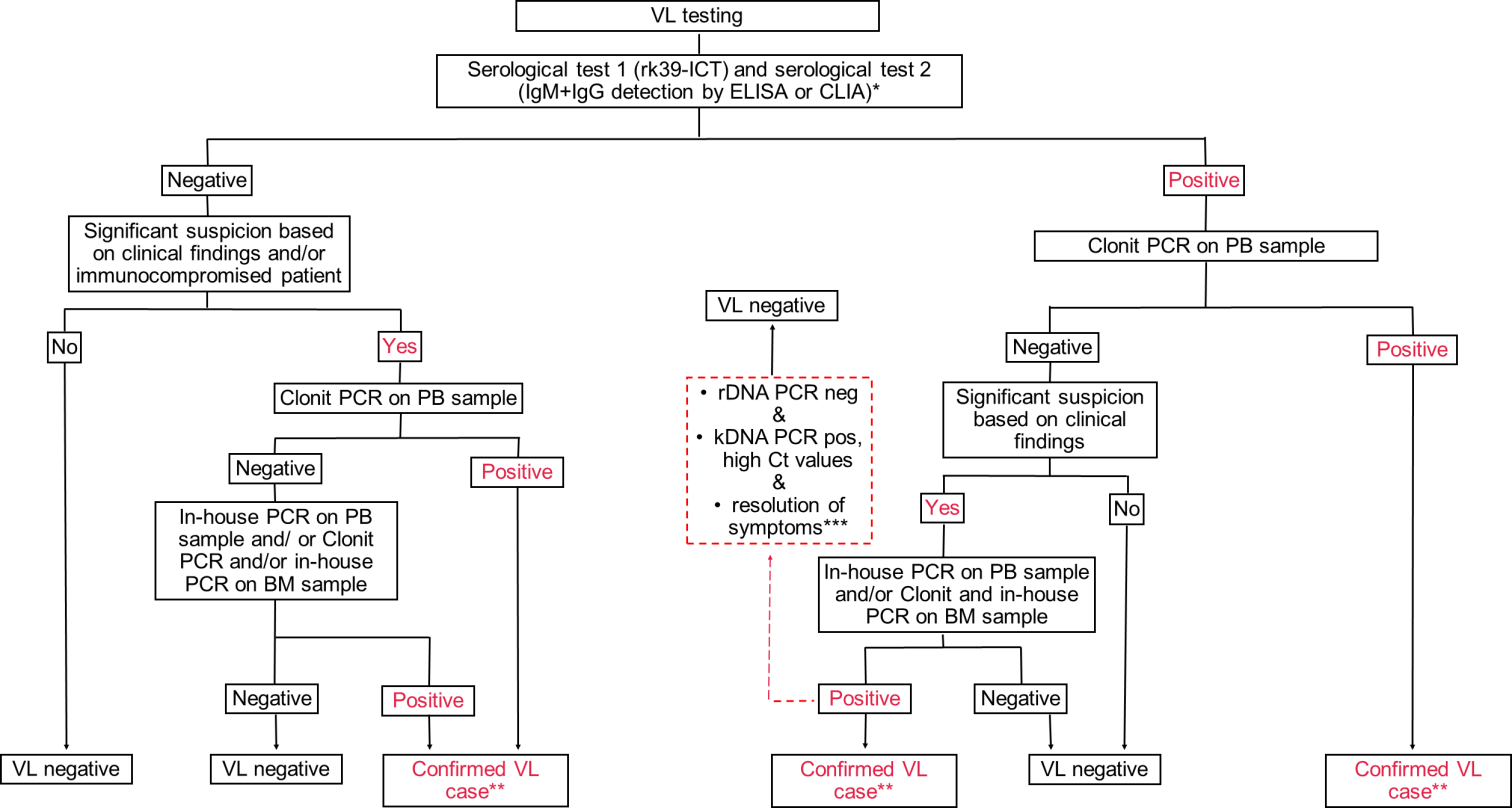
Diagnostic decision tree for visceral leishmaniasis (VL) applied by the reference laboratory for leishmaniasis of the Emilia-Romagna region, Northern Italy. rk39-ICT, immunochromatographic test based on rk39; ELISA, immunoenzymatic test; CLIA, chemiluminescent immunoassay; BM, bone marrow aspirate; PB, peripheral blood; kDNA, kinetoplast minicircle DNA; rDNA, 18S ribosomal RNA gene; Ct, cycle threshold; pos, positive; neg, negative. *In cases of significant clinical suspicion and/or critical clinical findings, serological testing and PCR are carried out concurrently; **Following clinical assessment by the infectious disease (ID) specialist; ***At follow-up assessment. Red dotted lines indicate exceptional cases.

The study includes two main parts. Firstly, we assessed the amplification performance of three real-time PCR assays using standardized *L. infantum* genomic DNA. In the second part, we evaluated the clinical performance of commercial versus in-house PCR assays on peripheral blood samples from patients with suspected VL. VL suspected cases were admitted at clinical centers in the Metropolitan Area of Bologna from August 2022 to July 2024. VL diagnosis was either confirmed or dismissed on the basis of molecular test results and clinical data (Fig. 1).

### Samples and data collection

Two types of samples were included in this study: standardized *L. infantum* genomic DNA and peripheral blood samples from patients with suspected VL. Standard samples were prepared using a *L. infantum* reference strain (MCAN/ES/98/LLM-877), cultured at 26°C *in vitro* as promastigotes in a custom-made liquid medium (HOMEM, Gibco Thermo Fisher Scientific Inc., Waltham, USA). The medium was enriched with 20% fetal bovine serum (EuroClone SpA, Milan, Italy) and 1% penicillin-streptomycin (EuroClone SpA). Parasites were harvested at the concentration of 10⁶ parasites/mL. For the second part of the study, a total of 90 blood samples were included. Collected data were recovered from the diagnostic activities of the RRL and organized in an electronic database by using a password-protected platform. Patients’ personal data were anonymized by an alphanumerical code. The study was carried out in compliance with the Declaration of Helsinki, and the protocol received approval from the Ethics Committee of the Area Vasta Emilia Centro (study ID: n° EM414-2023_97/2017/O/Tess/AOUBo). Samples were stored at −80°C before DNA extraction. *Trypanosoma cruzi* DNA (Tulahuen strain, TcVI) was kindly provided by Dr. Flores-Chávez (National Center for Microbiology, Instituto de Salud Carlos III, Spain) and tested at the concentration of 1x 10^6^ parasite equivalent/mL.

### Molecular standard and generation of calibration curves

To generate standard curves for the two in-house PCR assays as well as for the Clonit PCR assay, standardized *L. infantum* genomic DNA was extracted by using the semiautomated Maxwell CSC Instrument (Promega, Wisconsin, USA). Parasite quantification was accomplished by generating a standard curve, which consisted of serial 1:10 dilutions of *L. infantum* DNA ranging from the equivalent of 10^6^ to 10^−2^. For inter-assay validation, we carried out three independent repetitions of the entire experiment to generate the calibration curve, including separate DNA extractions from the parasite strain and independent amplification runs performed in different days. The resulting cycle threshold (Ct) values were analyzed using GraphPad Prism 9.5.1 (19) to generate a calibration curve based on the equation *y =* mx *+* q, where *y* represented the Ct value and x corresponded to the logarithm of the parasite concentration. The parasitic load was expressed as parasite equivalent/mL, representing the estimated number of parasites in a sample, calculated as the amount of parasitic DNA equivalent to one parasite whole genome per milliliter of blood (20). This allowed the determination of analytical parameters, including the limit of detection (LOD), the amplification efficiency, and the linearity range. In addition, the concentration of parasitic DNA in clinical samples was estimated by interpolating the Ct values.

### VL diagnosis

#### Diagnostic algorithm for VL

Laboratory testing for VL was performed at the RRL. According to the World Health Organization, there is no gold standard to identify VL, and the employment of a combination of indirect and direct tests is recommended to increase the diagnostic accuracy (2). A combination of two serological tests was employed to screen patients with suspicion of VL (7). Antibody-positive patients as well as antibody-negative patients that were immunocompromised and/or highly suspected for VL were tested by real-time PCR. All the steps are described in detail in the decision tree reported in Fig. 1. At our center, to confirm the VL diagnosis, both the following criteria had to be fulfilled: (i) clinical, biochemical, and hematological findings consistent with VL. Clinical signs suggestive of VL included prolonged irregular fever of unknown origin, splenomegaly, hepatomegaly, and loss of weight, while laboratory findings included anemia, thrombocytopenia, leukopenia, and hypergammaglobulinemia. The presence of hemophagocytic syndrome was also considered a suggestive sign of VL. (ii) A positive *Leishmania* real-time PCR result (either in-house PCR or Clonit PCR) in peripheral blood and/or bone marrow aspirate. Eligible participants met the following inclusion criteria: age between 0 and 99 years, clinical suspicion of VL, and provision of signed informed consent by patient or patients’ parents. For each patient, 5 mL of peripheral blood was collected.

#### Serological tests

Serological diagnosis was performed using a combined approach: (i) a rapid rK39-based immunochromatographic test (ICT, Leishmania Dipstick Rapydtest Apacor, Berkshire, England) that qualitatively detects antibodies against the *Leishmania* antigen rK39 and (ii) an immunoenzymatic test (Leishmania ELISA, Vircell, Granada, Spain) that was replaced in June 2023 by a chemiluminescence immunoassay (CLIA) for specific IgG and IgM detection (Leishmania VIRCLIA IgG + IgM monotest, Vircell).

#### In-house real-time PCR assays

To perform the in-house PCR assays, DNA extraction from clinical samples was performed by using the Maxwell CSC Blood DNA Kit (Promega, Wisconsin, USA), following the manufacturer’s recommendations and employing the semiautomated Maxwell CSC instrument (Promega, Wisconsin, USA). The procedure required 200 µL of whole blood, and DNA was eluted in 80μL of elution buffer. Five µL of extracted DNA was used for each PCR reaction. Two real-time PCR assays were carried out simultaneously, by amplifying (i) a segment of kDNA (18) and (ii) a segment of rDNA (14). Primers used for rDNA amplification (U1: 5′-AAGTGCTTTCCCATCGCAACT-3′; U2: 5′-GACGCACTAAACCCCTCCAA-3′) and for kDNA amplification (RV1: 5′-CTTTTCTGGTCCTCCGGGTAGG-3′; RV2: 5′-CCACCCGGCCCTATTTTACACCAA-3′) were synthesized by PrimmBiotech (Milan, Italy). Each PCR assay employed 50 pmol of TaqMan hydrolysis probes (FAM-CGGTTCGGTGTGTGGCGCC-TAMRA for rDNA; FAM-TTTTCGCAGAACGCCCCTACCCGC-TAMRA for kDNA), amplification was performed using the QuantStudio Dx Real Time PCR detection system (Thermofisher Scientific, Waltham, MA USA), and in-house kDNA and rDNA primers and probes were provided by Integrated DNA Technologies—IDT (Leuven, Belgium). Real-time PCR reactions were carried out by using 5 μL of extracted DNA in 25 μL total reaction volume in 1× PREMIX Ex Taq Perfect Real Time (Takara Bio Europe, Saint-Germain-en-Laye, France). A real-time PCR assay targeting the human β2-microglobulin gene was concurrently conducted to verify the quality and amplification efficiency of the extracted DNA. Thermal cycling conditions for amplification were as follows: initial denaturation at 95°C for 2 min, 1 cycle of 95°C for 15 s, 45 cycles of 60°C for 1 min. A tube of no-template control (NTC) was added to each PCR run; the run was considered valid if no amplification occurred in the NTC.

#### Clonit real-time PCR

Nucleic acid extraction was carried out using the ELITe InGenius total nucleic acid extraction kit (ELITechGroup Molecular Diagnostics, Turin, Italy) on ELITe InGenius SP200 instrument (ELITechGroup Molecular Diagnostics, Turin, Italy). DNA was eluted in 100 μL of elution buffer, and PCR was performed using the Leishmania spp Clonit RT-63 kit (CLONIT Srl Molecular Diagnostic, Milan, Italy), a CE-marked diagnostic assay that targets a segment of rDNA. The PCR runs were performed on ELITe InGenius SP200 instrument according to the manufacturer’s recommendations. The PCR amplification process included 45 cycles with 15 s at 95°C and 60 s at 60°C.

### Analysis of performances and statistical analysis

Analytical performances of the different PCR assays were evaluated by determining the linear dynamic range of the standard curve, calibration curve plotting, and interpolation. The limit of detection (LOD_95_) was defined as the lowest concentration of parasite genomic equivalents at which 95% of the standard replicates were successfully amplified (21). LOD_95_ was determined from three independent experiments, each including separate DNA extractions from the parasite strain and independent amplification runs performed on different days. Calibration curves were created by plotting Ct values against the logarithmic concentration of parasites by using GraphPad Prism 9.5.1 (19). These curves were also used to determine the PCR efficiency (Eff) and the linearity range for each assay. Eff was calculated based on the slope of the standard curve using the following formula Eff = 10^(−1/slope)^ − 1. Coefficient of determination (*R*^2^) was obtained by interpolating calibration data of each standard curve and represented the fit of the calibration curve with linear model. The precision of each molecular test was evaluated by assessing the inter-assay (inter-run) variation of Ct values obtained for the dilution series in three independent runs. The coefficient of variation (CV) was calculated as 100 × (standard deviation (SD) of Ct values from the three runs/mean Ct of the three runs). To evaluate analytical specificity, DNA extracted from a *T. cruzi* strain (Tulahuen strain, TcVI) was tested by employing the three PCR assays at 1 × 10^6^ parasites/mL concentration. Furthermore, analytical specificity was evaluated *in silico* using the primer-BLAST tool to verify the presence of the specific target sequence within the *L. infantum* reference genome (taxid: 5671) and its absence in the genomes of closely related human parasitic pathogens such as *T. cruzi* (taxid: 5693) and *T. brucei* (taxid: 5691). The potential cross-reactivity with the human genome was also assessed by evaluating the presence of the target sequence within the *Homo sapiens* genome (taxid: 9606).

Clinical sensitivity and specificity of the different molecular assays with their relative 95% confidence interval (CI) were calculated using classical 2 × 2 table analysis in MedCalc Software Ltd (22), based on the World Health Organization algorithm as reference. Concordance between the PCR assays was assessed using Cohen’s Kappa coefficient (κ). Results were interpreted according to the following κ values: (i) 0.01–0.20, slight agreement; (ii) 0.21–0.40, fair agreement; (iii) 0.41–0.60, moderate agreement; (iv) 0.61–0.80, substantial agreement; and (v) 0.81–1.00, excellent agreement. Differences of Ct values obtained by the amplification of clinical samples with the three real-time PCR assays were analyzed by using the pairwise Wilcoxon test. Comparison between quantitative data describing groups of samples amplified by different molecular assays was performed by using the Mann-Whitney *U* non-parametric test. For testing the goodness-of-fit of linear regression, the coefficient of determination *R*^2^ was used. When applicable, median, mean, and SD were calculated. Results were considered significant for *P*-values less than 0.05.

As the clinical diagnosis of VL cannot be assumed to be perfect (i.e., it is not a gold standard), we also analyzed the qualitative results from the three PCR assays using a latent-class model. For this analysis, we estimated the sensitivity and specificity of all three tests simultaneously without the need for a gold standard, using the Hui-Walter framework (23). In order to improve the analytical ability of the model, we split the data into two synthetic populations of roughly equal size by year of sampling: 2024 (*n* = 46) vs 2022–2023 (*n* = 44). The three-test, two-population model was implemented using Bayesian Markov chain Monte Carlo in JAGS (24) using the template_huiwalter function of the runjags package (25) for R (26) as an interface. Minimally informative Beta(1, 1) priors were used for all four sensitivity, four specificity, and two prevalence parameters within the model, and correlation terms were fitted for both sensitivity and specificity of the two rDNA-based tests to account for conditional dependence due to the similar analytical target. The model was run for 20,000 iterations (following a 5,000 iteration burn-in period) and was deemed to have converged by visual inspection of trace plots, verifying that the Gelman-Rubin statistic was below 1.05 for all parameters and that the effective sample size exceeded 1,000 for all parameters (27). Fit of the model was evaluated using the “LPmf” diagnostic criterion described by Comin et al. (28).

## RESULTS

### Analytical performance and limit of detection of the PCR assays

**Fig 2:**
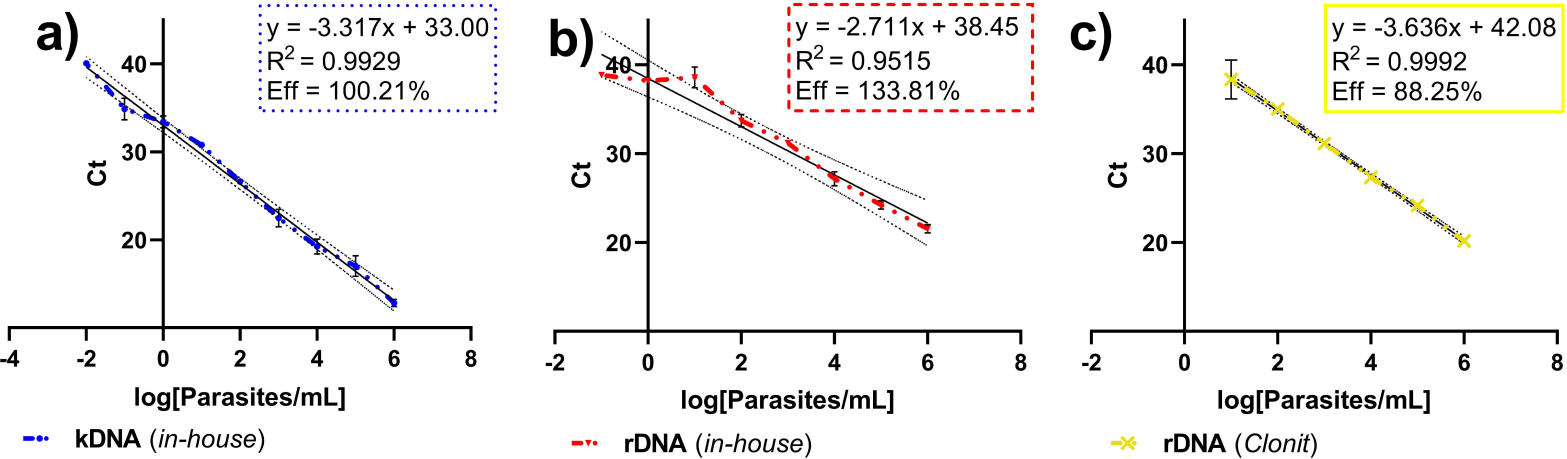
Calibration curves and real-time PCR efficiency estimation. Real-time PCR cycle threshold (Ct) values (*y*-axis) were obtained from amplification of serially diluted *L. infantum* standard samples (*x*-axis). Concentrations of standardized genomic DNA are plotted as the log₁₀ of the nominal values. The slope of the calibration curve was used to calculate PCR efficiency (Eff). (**a**) Kinetoplast minicircle (k)DNA PCR; (**b**): in-house 18S ribosomal RNA gene (rDNA) PCR; (**c**) Clonit rDNA PCR.

To evaluate the analytical performance of the molecular assays, serial 10-fold dilutions of cultured *L. infantum* promastigotes, ranging from 10⁶ to 10⁻² parasite equivalents/mL, were carried out and used to run the three PCR tests (Table 1). The kDNA PCR assay had the highest analytical sensitivity, with an Eff of 100.21% and the lowest LOD_₉₅_ value (1.01 × 10⁻¹ parasite equivalents/mL; Table 1 and Fig. 2a). The in-house rDNA assay achieved an Eff of 133.81% and a LOD_₉₅_ of 2.29 parasite equivalents/mL, while the Clonit PCR showed an Eff of 88.25% and a LOD_₉₅_ of 4.11 parasites/mL (Fig. 2b and c, respectively). Notably, the Clonit PCR maintained linear amplification through the entire tested concentration range (from 10^6^ to 10^−2^ parasite equivalents/mL), whereas the in-house rDNA assay displayed a more limited linear range (approximately 10^6^–10^3^ parasite equivalents/mL). The kDNA and the Clonit standard curve assays, but not the rDNA in-house PCR assay, demonstrated optimal linearity, with *R*² values exceeding 0.99. By evaluating the precision of the three PCRs, all the assays consistently exhibited low CV% values (<7%) at all standard curve dilutions, indicating low inter-assay variation (Tables S1 to S3).

**TABLE 1:** Calibration results obtained by the three real-time PCR based on standardized *L. infantum* genomic DNA^*b*^

	Parasites/mL	10^6^	10^5^	10^4^	10^3^	10^2^	10^1^	10^0^	10^−1^	10^−2^	LOD_95_ (parasite equivalents/mL)^*a*^
In-house kDNA	Amplified replicates (%)	3/3 (100%)	3/3 (100%)	3/3 (100%)	3/3 (100%)	3/3 (100%)	3/3 (100%)	3/3 (100%)	3/3 (100%)	1/3 (33.3%)	1.01 × 10^−1^

	Ct (mean ± SD)	12.82 ± 0.29	17.02 ± 1.64	19.24 ± 1.2	22.48 ± 1.24	26.6 ± 0.14	30.77 ± 0.01	33.41 ± 0.70	34.86 ± 1.65	40.05	
In-house rDNA	Amplified replicates (%)	3/3 (100%)	3/3 (100%)	3/3 (100%)	3/3 (100%)	3/3 (100%)	3/3 (100%)	1/3 (33.3%)	1/3 (33.3%)	0/3 (0%)	2.23

	Ct (mean ± SD)	21.53 ± 0.47	24.21 ± 0.46	27.16 ±0.8	31.19 ± 0.3	33.69 ± 072	38.56 ± 1.19	38.19	38.83	N/A	
Clonit rDNA	Amplified replicates (%)	3/3 (100%)	3/3 (100%)	3/3 (100%)	3/3 (100%)	3/3 (100%)	3/3 (100%)	0/3 (0%)	0/3 (0%)	0/3 (0%)	4.11

	Ct (mean ± SD)	20.01 ± 0.20	24.16 ± 0.14	27.32 ± 0.11	31.12 ± 0.15	35.02 ± 0.25	38.33 ± 2.20	N/A	N/A	N/A	

^*a*^ Limits of detection (LOD_95_), defined as the lowest concentration of parasite genomic equivalents at which 95% of replicates were successfully amplified, were calculated by interpolating the percentage of positive replicates at each dilution step using an asymmetric sigmoidal curve fitted in GraphPad Prism 9.5.1. Calculated data were based on three experimental replicates. Ct, cycle threshold; kDNA, kinetoplast minicircle DNA; N/A, no Ct values could be assigned; rDNA, 18S ribosomal RNA gene.

^*b*^ Standard samples used in the experiment were obtained from promastigotes of the reference strain JPCM5 resuspended in PBS.

The *in silico* analysis confirmed that the kDNA (RV1 and RV2) and rDNA (leishU1 and leishU2) primer pairs for the two in-house PCR matched their targets within the *L. infantum* genome, but not within *Trypanosoma* spp. or *H. sapiens* genomes, thus demonstrating the specificity of the primers. The primer-BLAST analysis is available at https://doi.org/10.6092/unibo/amsacta/8446. Finally, none of the three PCR assays produced amplification products with the *T. cruzi*-positive sample (data not shown), confirming the specificity of the three *Leishmania* PCR assays.

### Clinical performance of real-time PCR assays to diagnose VL

**Fig 3:**
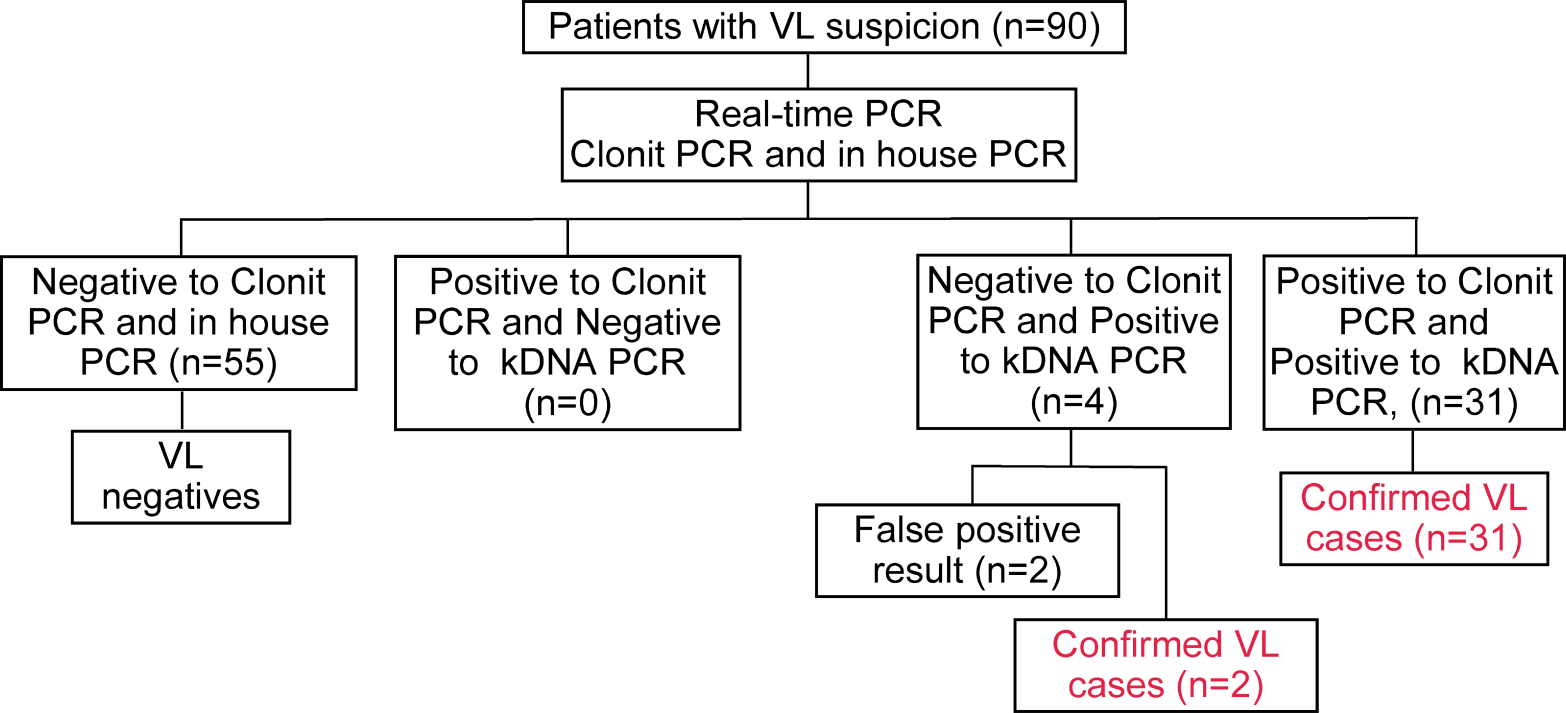
Study results based on diagnostic assays. Clonit PCR, commercial PCR assay based on amplification of the 18S ribosomal RNA gene ; kDNA PCR, in-house PCR based on amplification of the kinetoplast minicircle DNA; VL, visceral leishmaniasis.

A total of 90 peripheral blood samples were collected from an equal number of patients undergoing evaluation for suspected VL. Based on the diagnostic algorithm detailed in Fig. 1, 33 patients (36.7%) were confirmed as VL cases, while in 57 (63.3%) cases, VL diagnosis was dismissed (Fig. 3). Two patients were pediatric VL (aged 2 and 3 years, respectively), while all other VL cases occurred in adults. Serological and molecular tests employed in the study cohort as well as patient’s immune status are reported in Table S4.

The median age of the patient cohort was 58.5 years, with an age range of 1 to 90 years. Most patients were male (*n* = 72; 80%). Comparison of the Clonit PCR assay targeting rDNA with the reference diagnosis of VL exhibited two false-negative results, leading to a sensitivity of 93.94% (95% CI 79.77–99.26) and a specificity of 100% (95% CI 97.73–100.00) (Table 2).

**TABLE 2:** Clinical sensitivity and specificity of the three molecular assays evaluated for diagnosis of visceral leishmaniasis (VL)^*a*^

Molecular assay	Sensitivity (95% CI)	Specificity (95% CI)
Clonit (rDNA)	31/33 VL-positive, 93.94% (79.77%–99.26%)	0/57 VL-negative, 100.00% (93.73%–100.00%)
In-house (rDNA)	32/33 VL-positive, 96.97% (84.24%–99.92%)	0/57 VL-negative, 100.00% (93.73%–100.00%)
In-house (kDNA)	33/33 VL-positive, 100.00% (89.42%–100.00%)	2/57 VL-negative, 96.49% (87.89%–99.57%)

^*a*^ CI, confidence interval; kDNA, kinetoplast minicircle DNA; rDNA, 18S ribosomal RNA gene.

The in-house PCR targeting the rDNA achieved a sensitivity of 96.97% (95% CI 84.24–99.92) and a specificity of 100.00% (95% CI 93.73–100.00), closely aligned with the Clonit PCR. The in-house PCR targeting kDNA demonstrated a sensitivity of 100% (95% CI 89.42–100.00) but a specificity of 96.49% (95% CI 87.89%–99.57%), due to the presence of two false-positive cases. These two patients resided in the Bologna area. One patient was immunocompetent, while the other could be considered age-related immunocompromised, and, in both cases, kDNA PCR detected low levels of *Leishmania* DNA in peripheral blood (8.13 × 10^−3^, and 4.87 × 10^−3^ parasite equivalents/mL, respectively). In these two cases, *Leishmania* serology was positive by CLIA but negative by rk39 ICT in both patients (Table S4). Ultimately, VL was ruled out in both patients based on the following criteria: (i) negative *Leishmania* PCR result in bone marrow aspirate in one case, (ii) subsequent peripheral blood samples testing negative for *Leishmania* DNA between 1 and 9 months after the initial sampling, and (iii) spontaneous remission of symptoms.

In one case, kDNA PCR tested positive only in one out of three amplification replicates with high Ct value (40.6), while anti-*Leishmania* antibodies were not detected. Therefore, this case was classified as VL-negative, with the single positive PCR replicate likely attributable to contamination.

### Concordance between molecular methods

Concordance between the molecular assays was assessed by comparing the amplification results of all 90 clinical samples and calculating Cohen’s κ for each pairwise comparison. Comparison of the Clonit PCR with the in-house kDNA PCR revealed five discordant samples (two VL-positive, three VL-negative), yielding a κ = 0.90 (Table S5). The Clonit PCR and the in-house rDNA PCR showed one discordant sample, which was negative by the commercial PCR but positive by the in-house assay and diagnosed as VL-positive, resulting in a κ = 0.98 (Table S6). Comparing the two in-house PCR (rDNA vs kDNA), we identified three discordant cases, all positive by kDNA only (two VL-negative, one VL-positive), with a κ = 0.93 (Table S7).

### Agreement in quantitative results between real-time PCR assays

**Fig 4:**
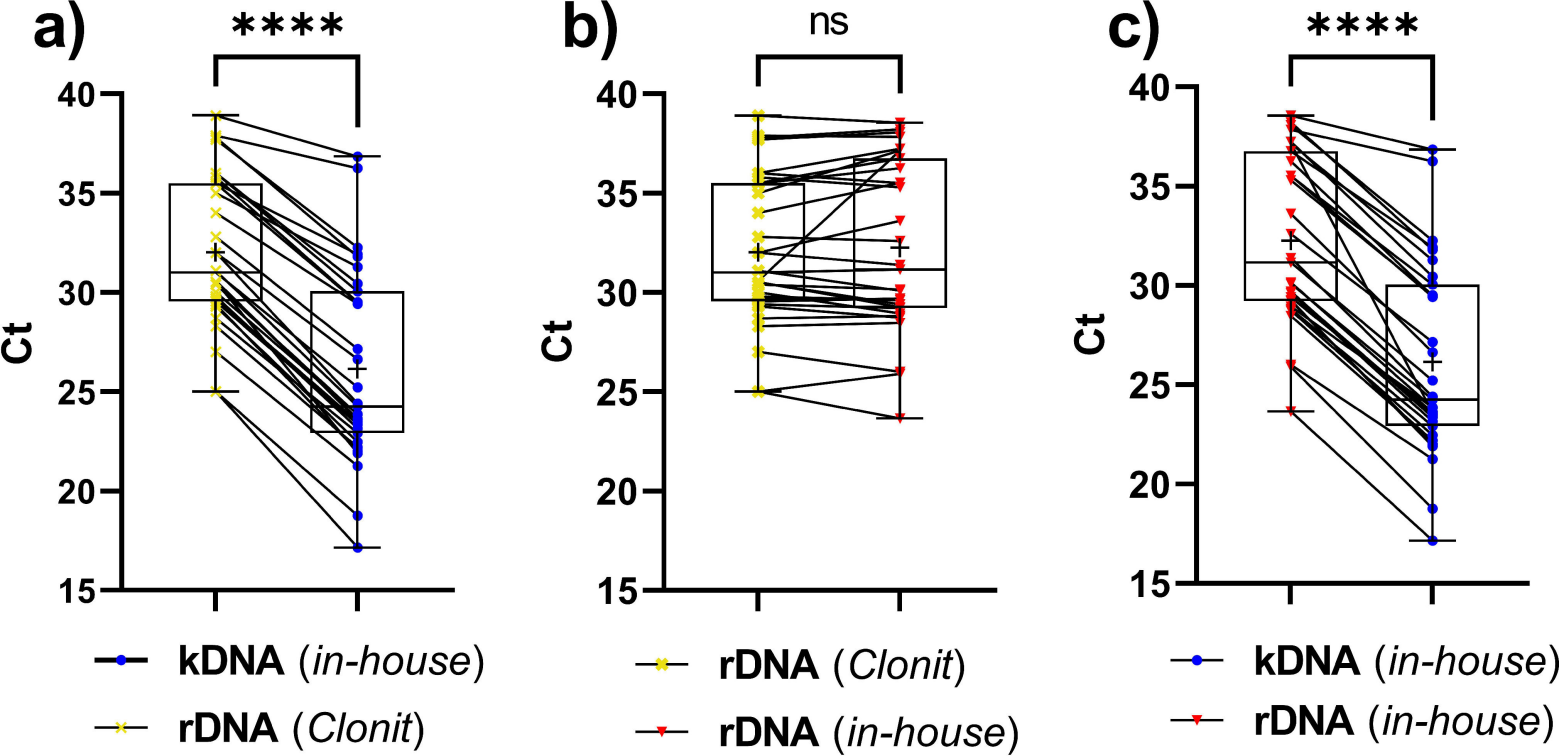
Pairwise cycle threshold (Ct) value comparison of real-time PCR assays for diagnosis of visceral leishmaniasis (VL). Thirty-one VL-positive samples were amplified by using the three PCR assays. Comparisons were performed between (**a**) in-house kinetoplast minicircle (k)DNA PCR and Clonit 18S ribosomal RNA gene (rDNA) PCR (pairwise Wilcoxon test *P*-value < 0.0001 [****]); (**b**) Clonit PCR and in-house rDNA PCR (pairwise Wilcoxon test *P*-value: 0.9961, not significant [ns]); (**c**) in-house kDNA PCR and in-house rDNA PCR (pairwise Wilcoxon test *P*-value < 0.0001 [****]). Data are presented as Tukey boxplots, where the box represents the interquartile range (from the first to the third quartile). The horizontal line within the box indicates the mean, while the cross depicts the median. Lines connecting paired boxplots represent values derived from the same sample tested with different molecular assays.

**Fig 5:**
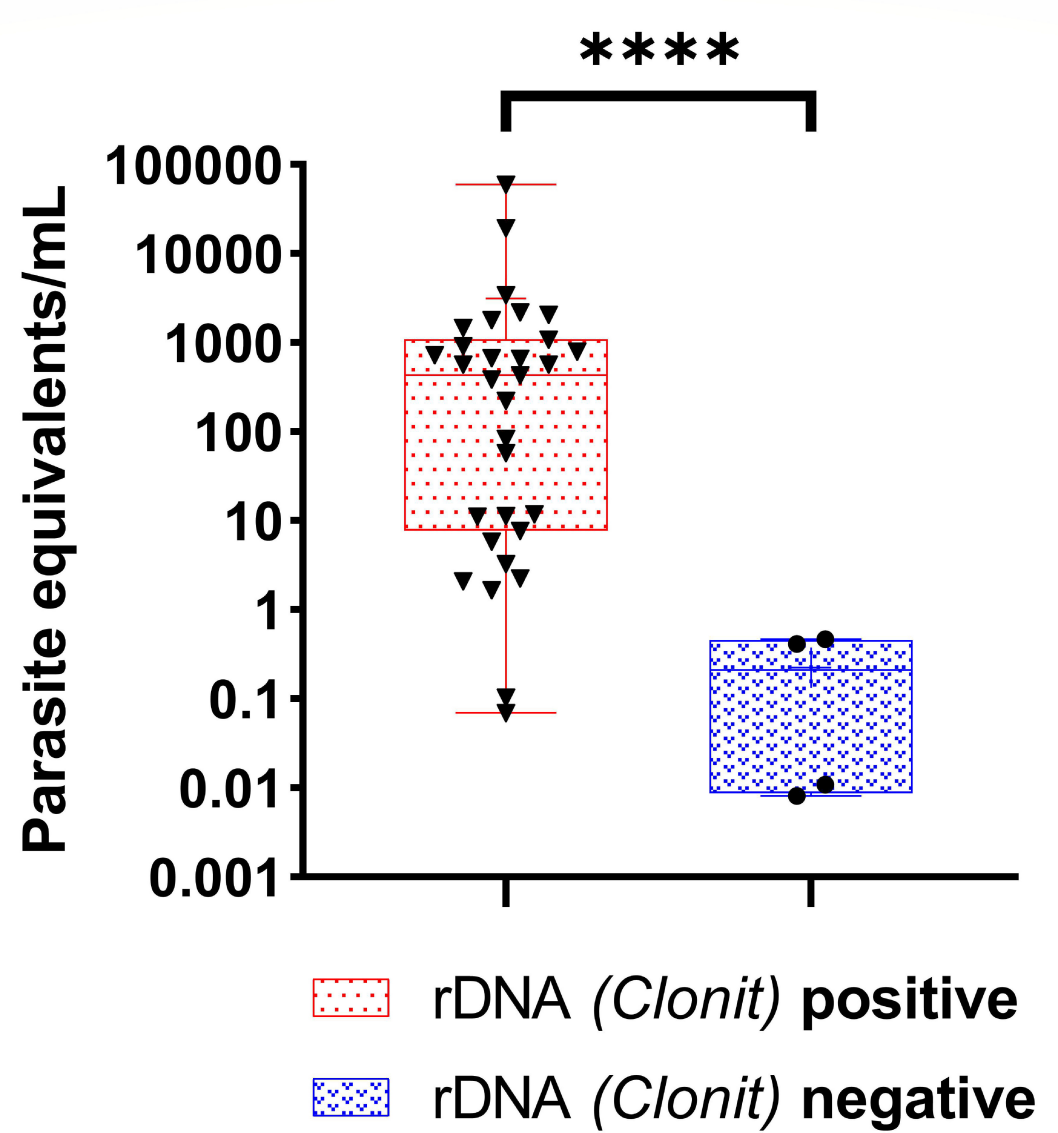
Relative concentration of parasite equivalents in samples from kinetoplast minicircle (k)DNA-positive cases stratified according to Clonit PCR results (*n* = 35). Distribution of relative parasitic DNA concentration (parasite equivalents/mL) in peripheral blood samples testing positive (red dotted boxes, *n* = 31, all visceral leishmaniasis [VL] positive cases) or negative (blue dotted boxes, *n* = 4, of them two were VL-positive cases, while two were VL-negative cases) by the commercial real-time PCR (Clonit), targeting the 18S ribosomal RNA gene (rDNA). The parasitic DNA concentration was calculated by interpolating Ct values from the in-house kDNA PCR assay using the standard calibration curve shown in Fig. 2. Statistical significance was assessed using the Mann-Whitney *U* test (*P* < 0.0005 [****]).

Ct values obtained from the amplification of 31 VL-positive samples, out of the total 33, tested positive by the 3 real-time PCR assays, were evaluated by pairwise comparison (Fig. 4). We observed a significant difference between the assays targeting rDNA and the assay targeting kDNA (*P* < 0.0001). Furthermore, we assessed the relative quantification of parasitic DNA per mL of blood in all kDNA PCR–positive samples, which included 33 VL-positive and 2 VL-negative cases. These samples were segregated into two groups: the first group comprised samples that tested positive by the Clonit PCR assay (*n* = 31, all from VL-positive patients), and the second group included samples that were negative by the Clonit PCR assay (*n* = 4, consisting of two VL-positive and two VL-negative patients; Fig. 5). We observed that the Clonit-positive group exhibited significantly higher concentration of parasitic DNA than the Clonit-negative group (*P*-value 0.0005). Descriptive statistics and distribution variability are summarized in Table 3.

**TABLE 3:** Cycle threshold (Ct) values and relative parasitic DNA concentration in kinetoplast minicircle (k)DNA-positive samples according to Clonit PCR results (*n* = 35)^*b*^

		Clonit PCR results			
		Positive, *n* = 31^*a*^		Negative, *n* = 4^*b*^	
		Ct (kDNA)	Parasite equivalents/mL^*c*^	Ct (kDNA)	Parasite equivalents/mL^*c*^
kDNA PCR results	Median	24.25	4.33 × 10^2^	36.90	2.11 × 10^−1^
	Mean	26.15	3.16 × 10^3^	36.96	2.25 × 10^−1^
	Minimum	36.85	6.93 × 10^−2^	39.93	8.13 × 10^−3^
	Maximum	17.15	6.01 × 10^4^	34.09	4.69 × 10^−1^
	Standard deviation	4.84	1.11 × 10^4^	3.21	2.50 × 10^−1^

^*a*^ 31 out of 31 cases were visceral leishmaniasis (VL)-positive.

^*b*^ 2 out of 4 cases were VL-positive.

^*c*^ estimated by using the real-time PCR targeting kDNA.

### Latent-class model results

Of the 90 clinical samples, 86 showed perfect concordance in qualitative result across the four tests (Table 4). Of the four samples that had imperfect agreement, two had a single positive (kDNA), one had two positive (kDNA and clinical diagnosis), and one had three positive results (all tests except Clonit). This reflects the excellent agreement between tests previously discussed. The latent-class model converged and showed no evidence of lack of model fit based on the LPmf criterion (data not shown). Sensitivity and specificity estimates obtained for each of the three tests from the latent-class model are shown in Table 5. As would be expected from the near-perfect agreement in qualitative results among the tests, the estimates were generally very similar between tests, although the median estimate for sensitivity was slightly lower for the Clonit PCR assay and that for specificity was slightly lower for the kDNA PCR test. The three molecular assays exhibited a high sensitivity (mean values >90%) in detecting VL cases. However, due to the limited amount of data available, the 95% credible intervals were relatively wide and overlapped substantially.

**TABLE 4:** Tabulated raw results of the three molecular assays^*a*^

Sampling year	Clonit rDNA PCR	In-house kDNA PCR	In-house rDNA PCR	n.
2022–2023	Neg	Neg	Neg	17
2024	Neg	Neg	Neg	38
2022–2023	Neg	Pos	Neg	2
2024	Neg	Pos	Neg	1
2022–2023	Neg	Pos	Pos	1
2022–2023	Pos	Pos	Pos	24
2024	Pos	Pos	Pos	7

^*a*^ kDNA, kinetoplast minicircle DNA; rDNA, 18S ribosomal RNA gene; n., number of cases; Neg, negative; Pos, positive.

**TABLE 5:** Posterior estimates of molecular diagnostic performance from the latent-class model^*a*^

Parameter	Test	Median	Lower 95% CI	Upper 95% CI	Mode	Mean	Prob. ≥90%	Prob. ≥95%
Sensitivity	Clonit rDNA PCR	93.36	84.04	99.25	94.66	92.56	0.76	0.33
Sensitivity	kDNA PCR	94.90	86.16	99.92	96.55	94.04	0.85	0.49
Sensitivity	in house rDNA PCR	95.24	87.21	99.91	96.62	94.44	0.88	0.53
Specificity	Clonit rDNA PCR	97.83	93.76	99.97	98.51	97.40	1.00	0.90
Specificity	kDNA PCR	92.40	84.90	98.18	93.13	91.95	0.74	0.21
Specificity	in house rDNA PCR	97.82	93.74	99.94	98.52	97.39	1.00	0.89

^*a*^ The table shows posterior median and 95% credible interval (CI) estimates for sensitivity and specificity of each of the tests (kDNA, kinetoplast minicircle DNA; rDNA, 18S ribosomal RNA gene), along with posterior mode and mean estimates for comparison. The right-hand two columns show the posterior estimate of the probability that the corresponding parameter exceeds a value of 90% and 95%, respectively.

## DISCUSSION

A variety of in-house assays have been developed for the molecular diagnosis of VL, with real-time PCR proposed as a potential future gold standard for *Leishmania* detection (29). However, only a few commercial PCR-based assays approved for *in vitro* diagnostic use are currently available. The rationale for this study was to generate comparative data on the analytical and clinical sensitivity of two in-house PCR and one commercial PCR assay for the diagnosis of VL.

In line with previous studies (17), in-house kDNA-targeting PCR demonstrated the highest analytical sensitivity, with an amplification efficiency of 100.21% and the lowest LOD_₉₅_ (1.01 × 10⁻¹ parasite equivalents/mL). This real-time PCR assay also exhibited the best clinical sensitivity. Among the rDNA-targeting assays, the in-house PCR and the Clonit PCR exhibited similar sensitivity both on standardized *L. infantum* genomic DNA and clinical samples, which demonstrates that the overall lower sensitivity of the rDNA-targeting assays is primarily attributable to the choice of the molecular target. In fact, the rDNA target is present at approximately 20–40 copies per parasite (14), while the kDNA is found in thousands of copies per parasite (18). On the other hand, the Clonit PCR exhibited the strongest reproducibility, likely due to its automated extraction and volume dispensing system. These features reduce the potential for human error and improve the reliability of parasite load quantification (30).

In terms of clinical performance, the three PCR assays exhibited high accuracy in the identification of VL, with both sensitivity and specificity exceeding 90%. Notably, the kDNA PCR assay achieved 100% sensitivity. Our findings are in line with those of a multi-center study involving reference laboratories in Europe, the United States, and Asia, which showed that kDNA-targeting PCR exhibited the highest sensitivity for detecting all *Leishmania* species when compared to assays targeting other genomic regions (31).

We also observed that the Clonit PCR failed in identifying two VL cases, exhibiting a sensitivity of 94%. The lower sensitivity of the commercial assay as compared to the kDNA PCR represents a significant limitation of the test, as the study cohort reflects the routine diagnostic practice and includes the range of parasite load typically found in clinical settings. Notably, the two false negatives of the Clonit PCR were identified as cases with a low concentration of parasitic DNA by the kDNA PCR assay (1.08 × 10^−1^ and 4.69 × 10^−2^ parasite equivalents/mL, respectively).

We also found that the kDNA-targeting PCR exhibited a specificity of 97%, with two false-positive results. These cases were characterized by very low concentrations of parasitic DNA (4.87 × 10^−3^, 8.13 × 10^−3^ parasite equivalents/mL, respectively). We previously observed low levels of parasitic DNA in a small number of asymptomatically infected individuals living in an endemic region (20). Therefore, the two VL false-positive results detected by kDNA PCR may reflect the detection of subclinical infections. Nevertheless, the possibility of PCR contamination cannot be ruled out; this risk could be considerably reduced by replacing the in-house assays with automated DNA extraction and PCR platform.

A study conducted in Colombia reported cross-amplification of the kDNA primers with *T. cruzi* and *Mycobacterium tuberculosis* DNA (32), underscoring the limitation of kDNA targeting assays in terms of specificity. However, it is important to note that the assay tested by Leon et al. employed a SYBR Green–based approach. Although this method is less expensive—and therefore more accessible in low- and middle-income countries—compared to those using Taqman or other oligonucleotide probes, this approach exhibits an increased risk of non-specific amplification, as previously demonstrated by Weirather et al. (12) for the same kDNA primer set. In line with these findings, when we tested *T. cruzi* DNA with the three *Leishmania* PCR TaqMan-based assays, no amplification products were detected.

This study exhibits several limitations, the most important being the low number of VL-positive clinical samples included in the study, which may impair the generalizability of our findings. Furthermore, the lack of a gold standard for VL diagnosis hampers the accurate assessment of test sensitivity. We also acknowledge the limited availability of published data from the same geographic area, which restricts the evaluation of potential exposure to other kinetoplastids, including *Leishmania tarentolae* (33). Despite these study limitations, the three real-time PCR assays showed excellent concordance in VL detection, with Cohen’s Kappa values ranging from 0.90 to 0.98. Pairwise comparisons of Ct values among the 31 VL-positive samples demonstrated consistent relative trends among assays, further supporting the reliability of the examined PCR assays for molecular diagnosis of VL.

When evaluating diagnostic test accuracy in the absence of a gold standard, it is important to consider the latent class implicitly identified by the model (34). In our setting, the three diagnostic tests were DNA-based, which implies that the latent class of the model closely matches “presence of genetic material from the pathogen.” The estimated sensitivity of these tests can therefore be interpreted as the probability of positive test results conditional on the presence of genetic material from the pathogen, which we interpret as being synonymous with active infection. However, some caution is warranted when interpreting these results due to the limited amount of data available, which is reflected in the relatively wide 95% CI estimates for all parameters. Due to substantially overlapping 95% CI for both sensitivity and specificity among tests, it is not possible to irrefutably conclude that any of the molecular tests outperformed the others.

*L. infantum* is the established causative agent of leishmaniasis in southern Europe, and its role in causing VL in Northern Italy has previously been confirmed by Internal Transcribed Spacer 1 typing of VL blood samples (35). In this study, all samples were collected from patients residing in Bologna, Northern Italy, where a distinct *L. infantum/L. donovani* hybrid strain has recently been identified as the causative agent of VL (36). Considering the peculiarity of this strain, prospective studies in other geographical areas are needed to further validate our observations.

In conclusion, the molecular assays evaluated in this study demonstrated high accuracy for the diagnosis of *L. infantum* VL using peripheral blood samples. The in-house PCR targeting kDNA had the highest observed sensitivity when compared to the Clonit PCR (rDNA) and the in-house PCR targeting rDNA. This characteristic is crucial for identifying VL in patients with low concentration of parasitic DNA in the blood, as the risk of misdiagnosis could have severe consequences. In addition, the kDNA target and its corresponding primer set have demonstrated optimal detection performance when applied to innovative analytical platforms distinct from real-time PCR, as shown recently by our group and others (29, 37). On the other hand, the kDNA-targeting assay exhibited a good linearity range but slightly higher variability compared to the Clonit PCR. Therefore, we strongly advocate for the development of automated diagnostic tools including kDNA as target to improve the identification of VL caused by *L. infantum*.

## Data Availability

The data used in the figures and tables are available in the public repository (AMSActa) https://doi.org/10.6092/unibo/amsacta/8767.

## SUPPLEMENTAL MATERIAL

Tables S1 to S3 jcm.01084-25-s0001.docx Inter-assay variation of the in-house kDNA PCR assay.

Table S4 jcm.01084-25-s0002.xlsx Serological and molecular tests and patient's immune status.

Tables S5 to S7 jcm.01084-25-s0003.docx Comparison between the examined PCR assays.

ASM does not own the copyrights to Supplemental Material that may be linked to, or accessed through, an article. The authors have granted ASM a non-exclusive, world-wide license to publish the Supplemental Material files. Please contact the corresponding author directly for reuse.
