# Supplementary material for: Analytical and clinical performance of in-house and commercial real-time PCR assays for diagnosing L. infantum visceral leishmaniasis: a study from a hub center in Northern Italy
Source: J Clin Microbiol. 2026 Feb 13;64(3):e01084-25. doi: 10.1128/jcm.01084-25 (PMC12977531; doi:10.1128/jcm.01084-25)
Supplement: Tables S1 to S3 — Inter-assay variation of the in-house kDNA PCR assay. [file jcm.01084-25-s0001.docx]

**Table S1. Inter-assay variation of the in-house kDNA PCR assay (Ct values).**

| **Parasite load/ml** | **Assay 1**  **(Ct)** | **Assay 2**  **(Ct)** | **Assay 3**  **(Ct)** | **Mean** | **SD** | **CV %** |
| --- | --- | --- | --- | --- | --- | --- |
| 10^6^ | 13.23 | 12.42 | 12.82 | 12.82 | 0.40 | 3.15 |
| 10^5^ | 18.15 | 17.09 | 15.82 | 17.02 | 1.16 | 6.84 |
| 10^4^ | 20.06 | 19.31 | 18.36 | 19.24 | 0.85 | 4.43 |
| 10^3^ | 23.08 | 23.05 | 21.32 | 22.48 | 1.00 | 4.47 |
| 10^2^ | 26.87 | 26.26 | 26.68 | 26.60 | 0.31 | 1.17 |
| 10^1^ | 30.78 | 30.74 | 30.79 | 30.77 | 0.02 | 0.07 |
| 10^0^ | 33.65 | 33.91 | 32.66 | 33.41 | 0.66 | 1.98 |
| 10^-1^ | 35.82 | 35.27 | 33.48 | 34.86 | 1.22 | 3.51 |
| 10^-2^ | 13.23 | 12.42 | 12.82 | 12.82 | 0.40 | 3.15 |

kDNA, kinetoplast minicircle DNA. Ct, cycle threshold. SD, standard deviation. CV, coefficient of variation.

**Table S2. Inter-assay variation of the Clonit PCR assay (Ct values).**

| **Parasite load/ml** | **Assay 1**  **(Ct)** | **Assay 2**  **(Ct)** | **Assay 3**  **(Ct)** | **Mean** | **SD** | **CV %** |
| --- | --- | --- | --- | --- | --- | --- |
| 10^6^ | 19.93 | 20.21 | 20.32 | 20.15 | 0.20 | 1.00 |
| 10^5^ | 24.09 | 24.07 | 24.32 | 24.16 | 0.14 | 0.58 |
| 10^4^ | 27.41 | 27.36 | 27.20 | 27.32 | 0.11 | 0.40 |
| 10^3^ | 31.21 | 30.95 | 31.20 | 31.12 | 0.15 | 0.47 |
| 10^2^ | 35.28 | 34.90 | 34.88 | 35.02 | 0.23 | 0.64 |
| 10^1^ | 40.44 | 38.50 | 36.05 | 38.33 | 2.20 | 5.74 |
| 10^0^ | na | na | na | nd | nd | nd |
| 10^-1^ | na | na | na | nd | nd | nd |
| 10^-2^ | na | na | na | nd | nd | nd |

kDNA, kinetoplast minicircle DNA. Ct, cycle threshold. SD, standard deviation. CV, coefficient of variation.

na, not amplified. nd, not determined.

**Table S3. Inter-assay variation of the in house rDNA PCR assay (Ct values).**

| **Parasite load/ml** | **Assay 1**  **(Ct)** | **Assay 2**  **(Ct)** | **Assay 3**  **(Ct)** | **Mean** | **SD** | **CV %** |
| --- | --- | --- | --- | --- | --- | --- |
| 10^6^ | 21.59 | 21.03 | 21.97 | 21.53 | 0.47 | 2.19 |
| 10^5^ | 24.57 | 24.37 | 23.69 | 24.21 | 0.46 | 1.91 |
| 10^4^ | 27.66 | 26.24 | 27.58 | 27.16 | 0.80 | 2.95 |
| 10^3^ | 31.45 | 31.26 | 30.87 | 31.19 | 0.30 | 0.95 |
| 10^2^ | 34.25 | 32.88 | 33.93 | 33.69 | 0.72 | 2.12 |
| 10^1^ | 38.02 | 37.73 | 39.92 | 38.56 | 1.19 | 3.09 |
| 10^0^ | na | 38.19 | na | nd | nd | nd |
| 10^-1^ | na | 38.83 | na | nd | nd | nd |
| 10^-2^ | na | na | na | nd | nd | nd |

kDNA, kinetoplast minicircle DNA. Ct, cycle threshold. SD, standard deviation. CV, coefficient of variation.

na, not amplified. nd, not determined.
