# Supplementary material for: Analytical and clinical performance of in-house and commercial real-time PCR assays for diagnosing L. infantum visceral leishmaniasis: a study from a hub center in Northern Italy
Source: J Clin Microbiol. 2026 Feb 13;64(3):e01084-25. doi: 10.1128/jcm.01084-25 (PMC12977531; doi:10.1128/jcm.01084-25)
Supplement: Tables S5 to S7 — Comparison between the examined PCR assays. [file jcm.01084-25-s0003.docx]

**Table S5. Comparison between the in-house kDNA PCR assay and the Clonit PCR assay (Cohen's κ =0.90).**

|  |  | **Clonit PCR** | | |
| --- | --- | --- | --- | --- |
|  |  | **pos** | **neg** | **tot** |
| **In-house kDNA PCR** | **pos** | 31 | 4 | 35 |
|  | **neg** | 0 | 55 | 55 |
|  | **tot** | 31 | 59 | 90 |

kDNA. kinetoplast minicircle DNA; pos. positive; neg. negative; tot. total.

**Table S6. Comparison between the in-house rDNA PCR assay and the Clonit PCR assay (Cohen's κ = 0.98).**

|  |  | **Clonit PCR** | | |
| --- | --- | --- | --- | --- |
|  |  | **pos** | **neg** | **tot** |
| **In-house rDNA PCR** | **pos** | 31 | 1 | 32 |
|  | **neg** | 0 | 58 | 58 |
|  | **tot** | 31 | 59 | 90 |

rDNA. 18s ribosomal RNA gene; pos. positive; neg. negative; tot. total.

**Table S7. Comparison between the in-house kDNA PCR assays and the in-house rDNA PCR assay (Cohen's κ = 0.93).**

|  |  | ***in-house* rDNA PCR** | | |
| --- | --- | --- | --- | --- |
|  |  | **pos** | **neg** | **Tot** |
| **in-house kDNA PCR** | **pos** | 32 | 3 | 35 |
|  | **neg** | 0 | 55 | 55 |
|  | **tot** | 32 | 58 | 90 |

kDNA. kinetoplast minicircle DNA; rDNA. 18s ribosomal RNA gene; pos. positive; neg. negative; tot. total.

Patients with VL suspicion

Serological test 1 (rk39-ICT) and serological test 2

(IgM+IgG detection by ELISA or CLIA)

Confirmed VL case

Strong clinical suspicion?

Positive

Negative

Positive

Negative

Real-time PCR

Clonit and/or in house on PB sample

Strong clinical suspicion?

Yes

No

False positive case by PCR

Strong clinical suspicion?

Yes

VL negative

No

- PCR on BM sample
- PCR on a second PB sample

Yes

VL negative
